# Reply to “Zimmermann, T., Lopez-Ayala, P. & Singer, M. Serial assessments of cardiac output and mixed venous oxygen saturation in comatose patients after out-of-hospital cardiac arrest”

**DOI:** 10.1186/s13054-023-04768-0

**Published:** 2023-12-14

**Authors:** Johannes Grand, Christian Hassager, Jesper Kjaergaard, Jacob E. Møller

**Affiliations:** 1 Department of Cardiology B, Section 2142, Copenhagen University Hospital, Rigshospitalet, Blegdamsvej 9, 2100 Copenhagen, Denmark; 2 Department of Clinical Medicine, University of Copenhagen, 2100 Copenhagen, Denmark; 3 Department of Cardiology, Odense University Hospital, 5000 Odense, Denmark; 4 Clinical Institute University of Southern, Odense, Denmark

Dear Editor,

We appreciate the opportunity to respond to the letter by Zimmermann et al. regarding our recent publication in Critical Care [1]. We are grateful for the interest in our work and acknowledge the importance of their queries and suggestions. Below, we address each point raised:

## Clarification on cox proportional hazards model

Our study utilized both univariable and multivariable Cox proportional hazards models as illustrated in Table 2. This table was supported by smoothing splines based on the univariable hazard model for illustration of the relationship between mortality and SvO_2_ and CI. We apologize for any lack of clarity in our manuscript and appreciate this opportunity to elucidate.

## Hazard ratio plot interpretation

Zimmermann et al. correctly note that our hazard plot in Fig. 4 did not specify a predefined reference point, making it a relative hazard plot. In retrospect, a dose–response plot with a clinically justified reference point would have provided good insights; however, choosing one over the other is a matter of debate.

Dose–Response Plots with a predefined reference can be intuitive and the reference point serves as a familiar benchmark. However, the choice of a reference point might introduce bias, especially if the reference point is not well-established. In this case, an appropriate cardiac index for comatose cardiac arrest patients is difficult to define. Furthermore, the analysis is somewhat anchored to the chosen reference point, which might limit exploring and understanding the broader context or relationships in the data.

Choosing the best plot for illustration purposes is important; however, we disagree that it could affect the conclusions drawn, since we based our conclusions on the Cox regression model as shown in Table 2. The splines figures are used as supportive illustrations only. The critique regarding the use of splines is well-taken. Our initial rationale for employing splines in the graphical illustration but not in the regression model was to maintain simplicity and interpretability in the model. However, we recognize that this may have led to a simplified representation of the data's complexities.

## Linearity assumptions

For the variable cardiac index, the variable did not satisfy the Cox regression models assumption of linearity. We therefore provided additional analysis with categorized variables quartiles. We acknowledge that this method has some drawbacks, especially in the form of loss of information when transforming a continuous variable into a categorical. However, this method does not compromise the assumptions for the Cox regression model. Furthermore, stratifying cardiac index into quartiles is clinically useful, since the highest and lowest quartile is the groups with highest risk of adverse outcomes from a clinical point of view.

In conclusion, we thank Zimmermann et al. for their constructive criticism, which has prompted a re-examination of our data and methodologies. We believe that the opportunity to address these points will not only strengthen the interpretation of our current study but also contribute to the broader discussion on the value of cardiac output and venous oxygen saturation in comatose patients following cardiac arrest.
